# Supplementary material for: Prognostic Value of the Serum HER2 Extracellular Domain Level in Breast Cancer: A Systematic Review and Meta-Analysis
Source: Cancers (Basel). 2022 Sep 20;14(19):4551. doi: 10.3390/cancers14194551 (PMC9559205; doi:10.3390/cancers14194551)

Supplementary Materials

Table S1. Full search strategy in PubMed (a), Embase (b), the Cochrane Library (c) Web of Science (d) and Scopus (e).
Table S2. Newcastle–Ottawa Quality Assessment form for non-randomized studies.
Figure S1. Sensitivity analysis for PFS(a), DFS(b), OS(c) and ORR(d).
Figure S2. Subgroup analysis of PFS according to different treatment modalities(a), cut-off values(b) and ethnicity(c).
Figure S3. Subgroup analysis of DFS according to different treatment modalities(a), cut-off values(b) and ethnicity(c).
Figure S4. Subgroup analysis of OS according to different treatment modalities(a), cut-off values(b), disease status(c) and ethnicity(d).
Figure S5. Subgroup analysis of ORR according to different treatment modalities(a), cut-off values(b) and ethnicity(c).
Figure S6. Funnel plot to detect publication bias for PFS(a), DFS(b), OS(c) and ORR(d).
Figure S7. Trim and fill analysis for pooled HR of PFS.

Supplementary Table S1 Detailed searching strategies.

a. Search strategy in PubMed, May 2nd, 2022. (n=758)

Search terms: ((("Breast Neoplasms"[Mesh])OR((breast OR mammary) AND (cancer OR carcinoma OR malignancy OR neoplasm OR tumor))) AND (("Receptor, ErbB-2"[Mesh])OR((HER-2 OR HER2 OR neu OR ERBB2 OR "human epidermal growth factor receptor 2")))) AND (("extracellular domain" OR ECD OR "soluble HER2" OR "serum HER2" OR sHER2))

b. Search strategy in Embase, May 2nd, 2022. (n=526)

Search terms:
#10 #3 AND #6 AND #9
#9 #7 OR #8
#8 'ecd':abti OR 'soluble her2':abti OR 'serum her2':abtiOR'sher2'abti
#7 extracellular domain'/exp
#6 #4 OR #5
#5 'her-2':ab.ti OR 'her2':ab.ti OR 'neu':ab.ti OR 'erbb2':abti OR 'human epidermal arowth factor receptor 2':ab.ti
#4 "epidermal growth factor receptor 2'/exp
#3 #1 OR #2
#2 ('breast':ab,ti OR 'mammary':ab,ti) AND ('cancer':abti OR 'carcinoma':ab,ti OR 'malignancy':ab,ti OR 'neoplasm':ab,ti OR 'tumor':abti)
#1 'breast cancer'/exp

c. Search strategy in the Cochrane Library, May 2nd, 2022. (n=97)

Search terms:
((("Breast Neoplasms"[Mesh])OR((breast OR mammary) AND (cancer OR carcinoma OR malignancy OR neoplasm OR tumor))) AND (("Receptor, ErbB-2"[Mesh])OR((HER-2 OR HER2 OR neu OR ERBB2 OR "human epidermal growth factor receptor 2")))) AND (("extracellular domain" OR ECD OR "soluble HER2" OR "serum HER2" OR sHER2))

d. Search strategy in the Web of Science, Sept 11th, 2022. (n=98)

Search terms:
#10 #8 AND #9 AND #6
#9 #4 OR #5
#8 #1 OR #7
#7 #2 AND #3
#6 ALL=("extracellular domain" OR ECD OR "soluble HER2" OR "serum HER2" OR sHER2)
#5 ALL=(HER-2 OR HER2 OR neu OR ERBB2 OR "human epidermal growth factor

receptor 2")
#4 TS="Receptor, ErbB-2"
#3 ALL=(cancer OR carcinoma OR malignancy OR neoplasm OR tumor)
#2 ALL=(breast OR mammary)
#1 TS=Breast Neoplasms

e. Search strategy in the Scopus, Sept 11th, 2022. (n=779)

Search terms:
((KEY ("Breast Neoplasms")) OR (TITLE-ABS-KEY ((breast OR mammary) AND (cancer OR carcinoma OR malignancy OR neoplasm OR tumor)))) AND ((KEY ("Receptor, ErbB-2")) OR (TITLE-ABS-KEY (her-2 OR her2 OR neu OR erbb2 OR "human epidermal growth factor receptor 2"))) AND (TITLE-ABS-KEY ("extracellular domain" OR ecd OR "soluble HER2" OR "serum HER2" OR sher2))

Supplementary Table S2. Newcastle-Ottawa Quality Assessment form for non-randomized studies.

Studies	Selection	Comparability	Outcome	Quality Assessment
Banys et al. 2017	❋ ❋ ❋ ❋	❋	❋ ❋	Good quality
Baric et al. 2015	❋ ❋ ❋	❋	❋ ❋	Poor quality
Bewick et al. 2001	❋ ❋ ❋ ❋	❋	❋ ❋ ❋	Good quality
Bramwell et al. 2009	❋ ❋ ❋ ❋		❋ ❋ ❋	Good quality
Colomer et al. 2007	❋ ❋ ❋ ❋		❋ ❋ ❋	Good quality
Colomer et al. 2004	❋ ❋ ❋ ❋	❋	❋ ❋ ❋	Good quality
Colomer et al. 2006	❋ ❋ ❋ ❋		❋ ❋ ❋	Good quality
David et.al. 2008	❋ ❋ ❋ ❋	❋	❋ ❋ ❋	Good quality
Darlix et al. 2016	❋ ❋ ❋	❋	❋ ❋ ❋	Good quality
Gioia et al. 2015	❋ ❋ ❋ ❋	❋	❋ ❋ ❋	Good quality
Eppenberger et al. 2020	❋ ❋ ❋ ❋	❋	❋ ❋ ❋	Good quality
Esteva et al. 2002	❋ ❋ ❋	❋	❋ ❋	Poor quality
Finn et al. 2009	❋ ❋ ❋ ❋	❋	❋ ❋ ❋	Good quality
Fornier et al. 2005	❋ ❋ ❋		❋ ❋ ❋	Good quality
Im et al. 2005	❋ ❋ ❋ ❋		❋ ❋ ❋	Good quality
Jensen et al. 2003	❋ ❋ ❋ ❋	❋	❋ ❋ ❋	Good quality
Knutson et al. 2014	❋ ❋ ❋ ❋		❋ ❋ ❋	Good quality
Kong et al. 2012	❋ ❋ ❋ ❋	❋	❋ ❋ ❋	Good quality
Kontani et al. 2013	❋ ❋ ❋ ❋		❋ ❋	Poor quality
Kostler et al. 2004	❋ ❋ ❋	❋	❋ ❋ ❋	Good quality
Lee et al. 2016	❋ ❋ ❋		❋ ❋	Poor quality
Lee et al. 2014	❋ ❋ ❋ ❋		❋ ❋ ❋	Good quality
Lipton et al. 2002	❋ ❋ ❋	❋	❋ ❋	Poor quality
Lipton et al. 2003	❋ ❋ ❋ ❋	❋	❋ ❋	Good quality
Lipton et al. 2011	❋ ❋ ❋ ❋		❋ ❋	Poor quality
Ludovini et al. 2008	❋ ❋ ❋ ❋	❋	❋ ❋ ❋	Good quality
Luftner et al. 2004	❋ ❋ ❋ ❋		❋ ❋	Poor quality
Molina et al. 2010	❋ ❋ ❋ ❋	❋	❋ ❋	Good quality
Moreno-Aspitia et al. 2013	❋ ❋ ❋	❋	❋ ❋ ❋	Good quality
Muller et al. 2004	❋ ❋ ❋	❋	❋	Poor quality
Reix et al. 2016	❋ ❋ ❋ ❋	❋	❋ ❋	Good quality
Ryu et al. 2012	❋ ❋ ❋		❋ ❋ ❋	Poor quality
Sandri et al. 2004	❋ ❋ ❋ ❋		❋ ❋ ❋	Good quality
Shao et al. 2014	❋ ❋ ❋ ❋		❋	Poor quality

Tchou et al. 2015	✵ ✵ ✵ ✵	✵	✵ ✵ ✵	Good quality
Thureau et al. 2012	✵ ✵ ✵ ✵	✵	✵ ✵ ✵	Good quality
Tsai et al. 2012	✵ ✵ ✵ ✵	✵	✵ ✵ ✵	Good quality
Wang et al. 2016	✵ ✵ ✵ ✵	✵	✵ ✵	Good quality
Witzel et al. 2006	✵ ✵ ✵ ✵	✵	✵ ✵ ✵	Good quality
Zuo et al. 2021	✵ ✵ ✵	✵	✵ ✵ ✵	Good quality

Supplementary Figure S1. Sensitivity analysis for PFS(a), DFS(b), OS(c) and ORR(d).

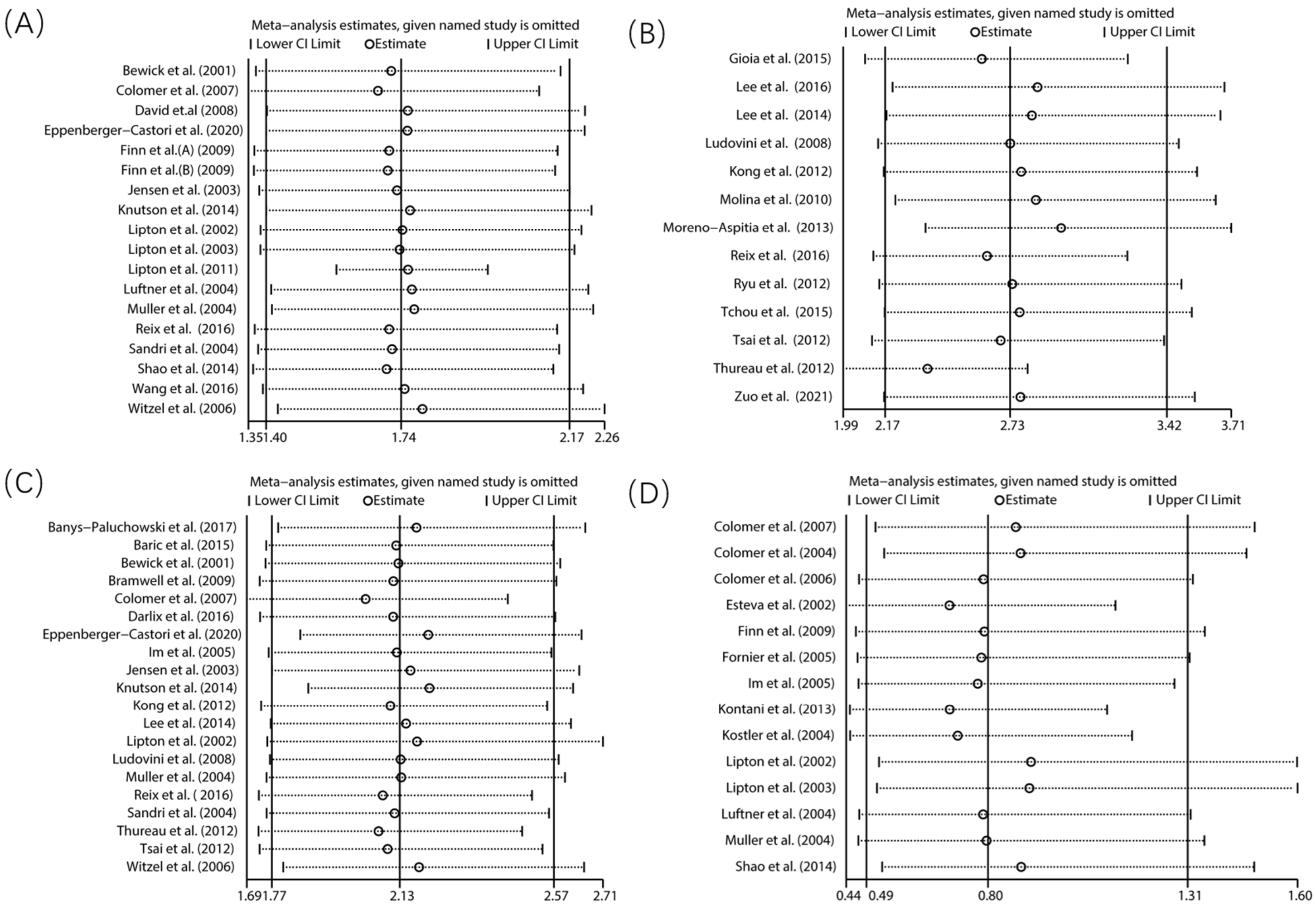

Supplementary Figure S2. Subgroup analysis of PFS according to different treatment modalities(a) and cut-off values(b).

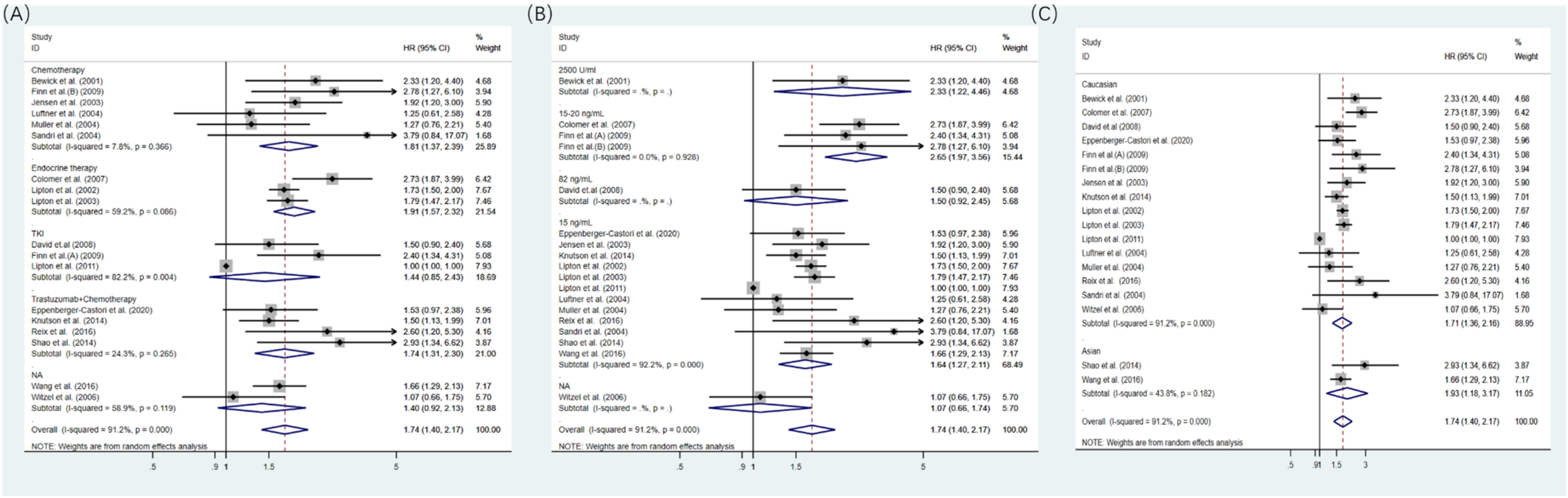

Supplementary Figure S3. Subgroup analysis of DFS according to different treatment modalities(a) and cut-off values(b).

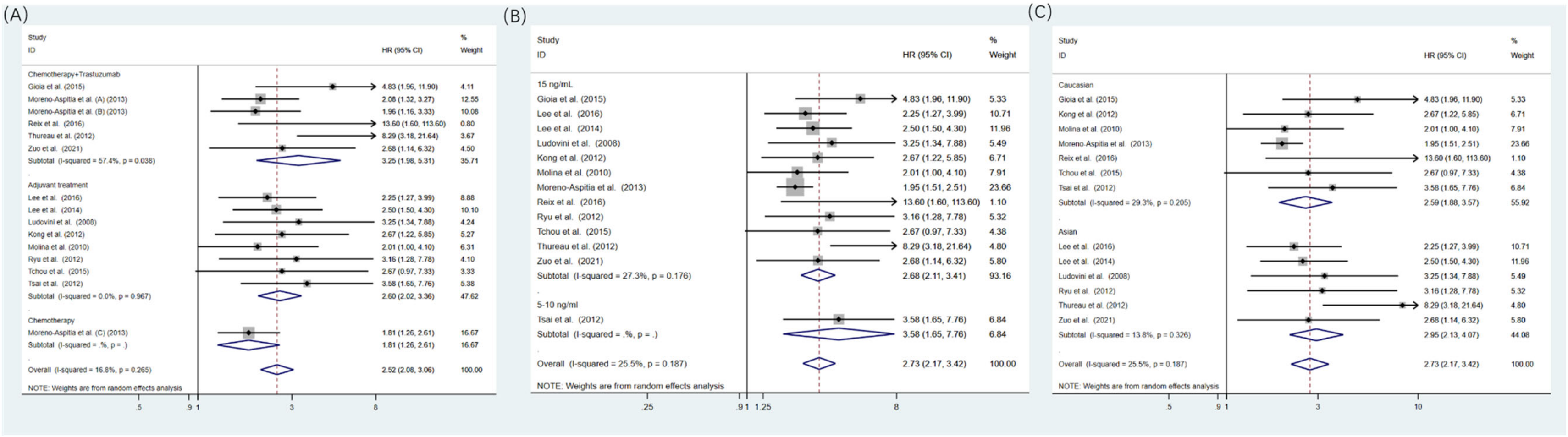

Supplementary Figure S4. Subgroup analysis of OS according to different treatment modalities(a), cut-off values(b), and disease status(c).

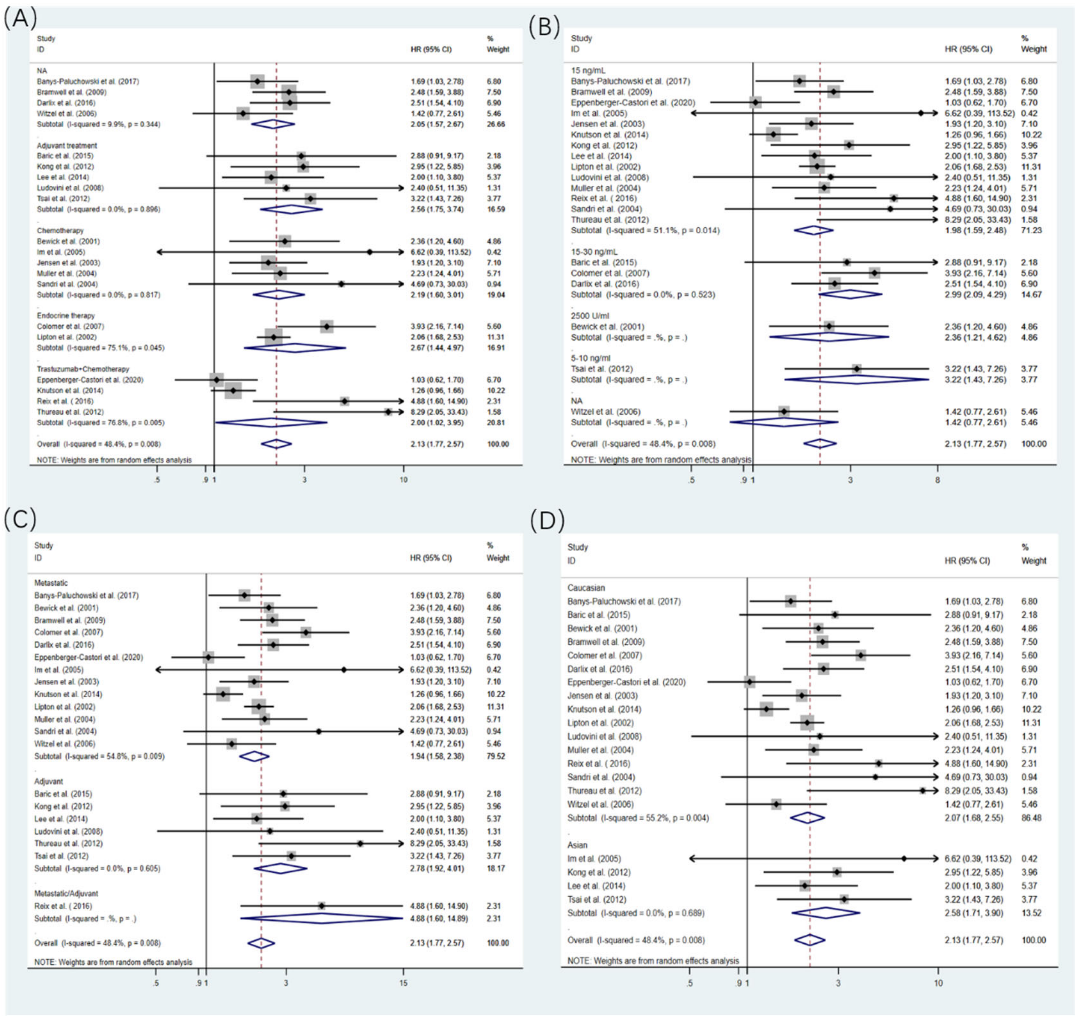

Supplementary Figure S5. Subgroup analysis of ORR according to different treatment modalities(a) and cut-off values(b).

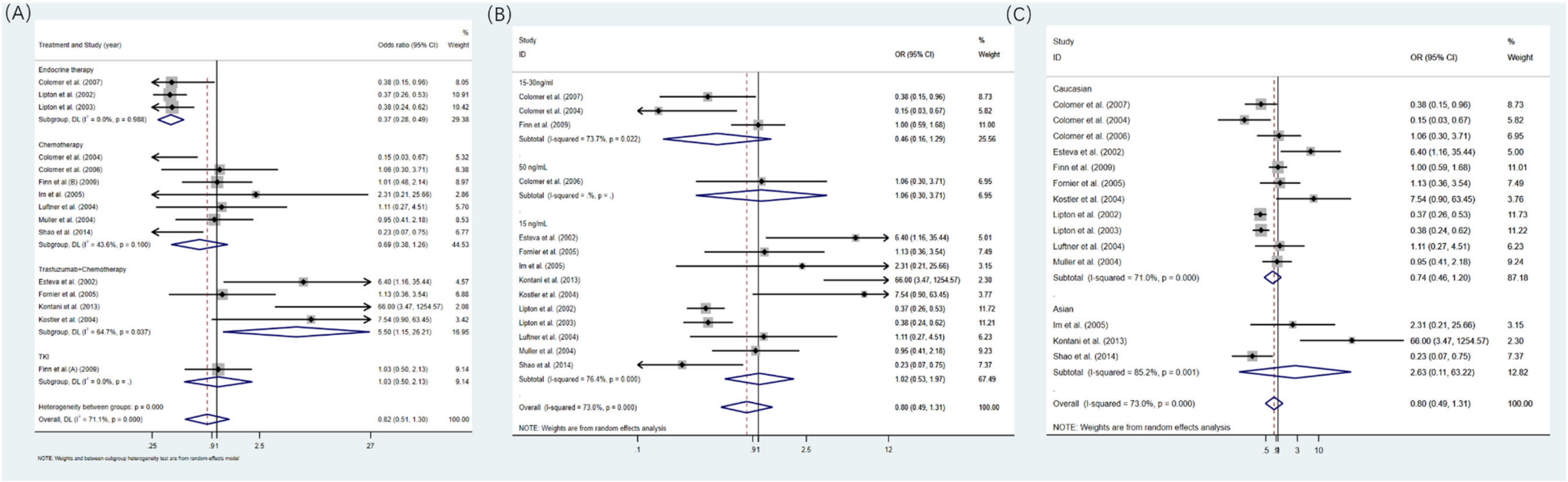

Supplementary Figure S6. Funnel plot to detect publication bias for PFS(a), DFS(b), OS(c) and ORR(d).

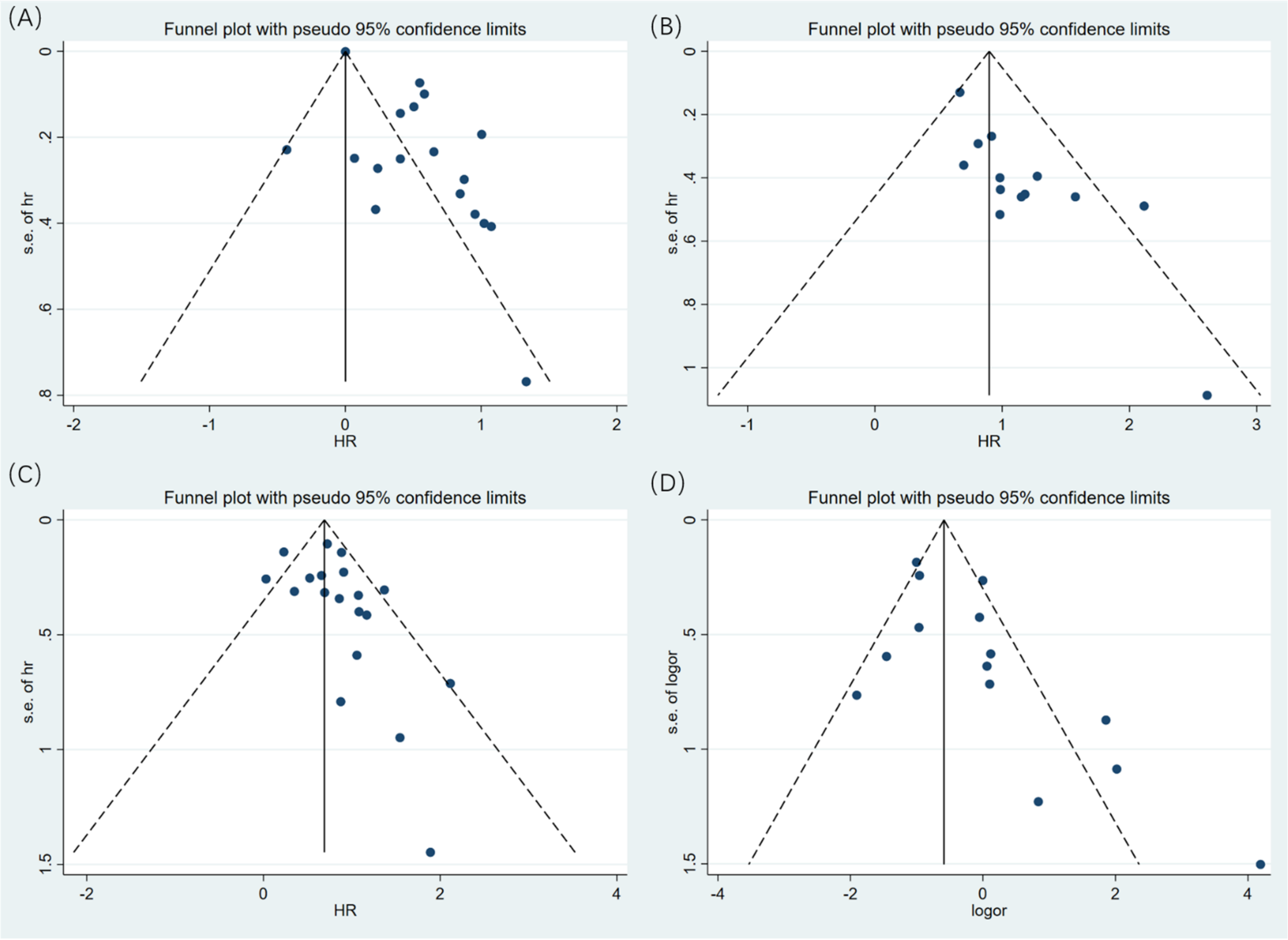

Supplementary Figure S7. Trim and fill analysis for pooled HR of PFS.

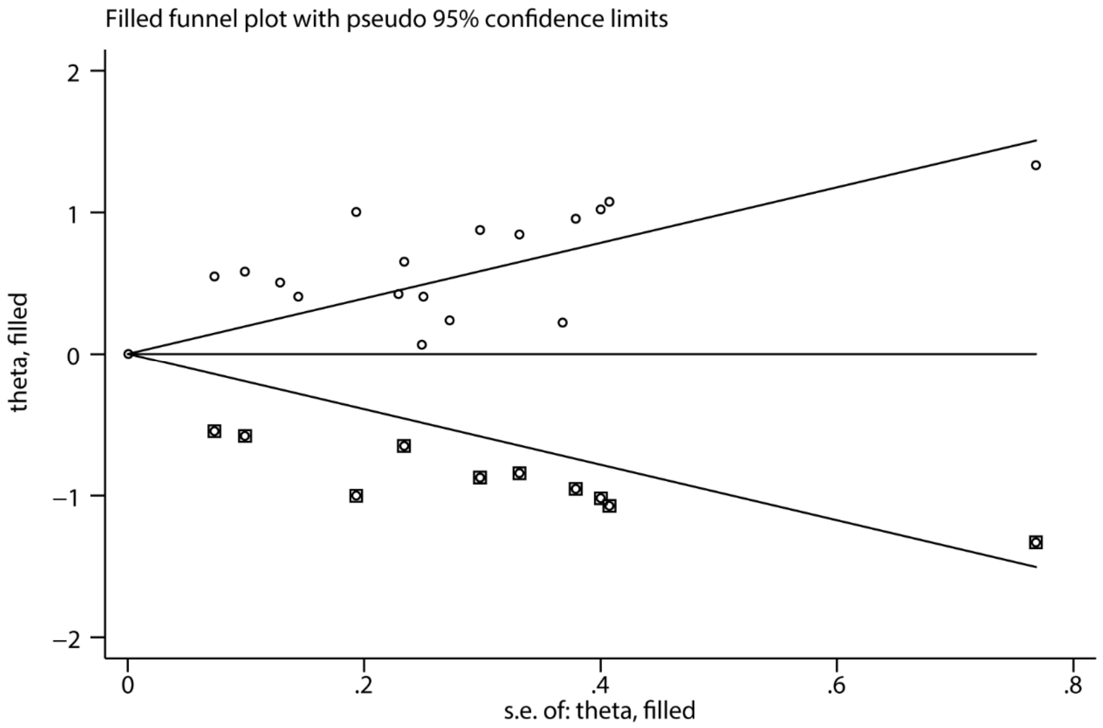

Supplement: Supplementary file 1 [file cancers-14-04551-s001.zip › cancers-1884311-supplementary.pdf]
